# Identifying risk factors for recurrent multidrug resistant tuberculosis based on patient’s record data from 2016 to 2021: retrospective study

**DOI:** 10.1038/s41598-024-73209-x

**Published:** 2024-10-13

**Authors:** Teramaj Wongel Wotale, Mesfin Esayas Lelisho, Bikiltu Wakuma Negasa, Seid Ali Tareke, Woldemariam Erkalo Gobena, Ebsa Gelan Amesa

**Affiliations:** 1 Department of Statistics, College of Natural and Computational Sciences, Dilla University, Dilla, Ethiopia; 2 Department of Statistics, College of Natural and Computational Sciences, Mizan-Tepi University, Tepi, Ethiopia; 3 Department of Statistics, College of Natural and Computational Sciences, Mattu University, Mattu, Ethiopia

**Keywords:** Recurrence, Retrospective, MDR-TB, Shared frailty, Gastrointestinal diseases, Infectious diseases, Reproductive disorders, Respiratory tract diseases, Immunology, Psychology, Diseases, Health care, Health occupations, Medical research, Risk factors, Signs and symptoms

## Abstract

Globally, the prevalence of multidrug-resistant tuberculosis (MDR-TB) has been increasing recently. This is a major public health concern, as MDR-TB is more difficult to treat and has poorer outcomes compared to drug-sensitive tuberculosis. The main objective of the study was to identify risk factors for recurrent multidrug-resistant tuberculosis, at Alert Specialized Hospital, Addis Ababa, by using different parametric shared frailty models. From January 2016 to December 2021, a retrospective study was conducted on MDR-TB patients at Alert Specialized Hospital in Addis Ababa. The data for the study were collected from the medical records of MDR-TB patients at the hospital during this time period. Gamma and inverse-Gaussian shared frailty models were used to analyze the dataset, with the exponential, Weibull, and lognormal distributions included as baseline hazard functions. The data were analyzed using R statistical software. The median recurrence time of the patients was 12 months, and 149 (34.3%) had recurrences. The clustering effect was statistically significant for multiple drug-resistant tuberculosis patients’ recurrence. According to the Weibull-Inverse-Gaussian model, factors that reduced time to MDR-TB recurrence included lower weight (ɸ = 0.944), smoking (ɸ = 0.045), alcohol use (ɸ = 0.631), hemoptysis (ɸ = 0.041), pneumonia (ɸ = 0.564), previous anti-TB treatment (ɸ = 0.106), rural residence (ɸ = 0.163), and chronic diseases like diabetes (ɸ = 0.442) were associated with faster recurrence. While, higher education (ɸ = 3.525) and age (ɸ = 1.021) extended time to recurrence. For weight increment, smokers and alcohol users, clinical complications of hemoptysis and pneumonia, patients with pulmonary disease who had a history of previous anti-TB treatment, and being rural residents are prognostic factors. There was a significant clustering effect at the Alert Specialized Hospital in Addis Ababa, Ethiopia. The Weibull-Inverse Gaussian Shared Frailty Model was chosen as the best model for predicting the time to recurrence of MDR-TB.

## Introduction

Multidrug-resistant tuberculosis (MDR-TB) is the term used to describe a strain of Mycobacterium tuberculosis that exhibits combined resistance to isoniazid and rifampin^1^. Many MDR-TB patients have been taking anti-TB medications for a long time, often irregularly, resulting in treatment failure^2^. It has also been demonstrated that people who have primary resistance and frequently fail treatment may become increasingly resistant and difficult to treat. Due to that patients with MDR-TB have poorer treatment outcomes than those with drug-sensitive TB^3,4^.

MDR-TB is a major concern on the international, national, and regional levels^5^. In 2022, approximately 10.6 million people were diagnosed with tuberculosis (TB). Of these, 3.3% were newly diagnosed with MDR-TB, while 17% had previously been treated for TB and were diagnosed with MDR-TB, resulting in approximately 410,000 new cases of MDR-TB^6^. According to the global TB report, 3.4% of MDR-TB cases were new, while 18% had previously been treated^7^. According to the World Health Organization (WHO), 484,000 new cases of rifampicin resistance were reported, and of those, 78% had MDR-TB^8^.

Recurrence following successful treatment of multidrug-resistant tuberculosis is not unexpected, according to a growing body of research conducted in the last few years^9^. Recurrence rates have been reported worldwide ranging from 0%^10^ following two years of follow-up to 8.5% following eight years of follow-up^11^. In China, MDR-TB was found in 98%of bacteriologically confirmed cases^12^. The African continent has the highest reported incidence rate of 475 cases per 100,000 people, accounting for 46% of all tuberculosis cases worldwide^13^.

Sub-Saharan Africa has a higher rate of tuberculosis infection, accounting for an estimated 24% of the 10 million cases of tuberculosis worldwide^14^. In Ethiopia, tuberculosis (TB) is the leading cause of death from infectious diseases, with 30,000 deaths each year^15^. Ethiopia has 2700 MDR-TB cases annually, making it one of the 30 countries in the world with the highest MDR-TB burdens^16^. The other studies also indicated that MDR-TB was highly prevalent in some areas of Ethiopia, including 11.8% in Tigray^17^,15.3% in Amhara^18^, and 46.3% in Addis Ababa^19^.

Even though a plan to offer drug susceptibility and cultural testing services has been discussed, many people’s lives are still being faced with great challenges on both a regional and national level in the nation^20^. Access to these services is often limited, especially in rural areas. This is due to several factors, including the lack of qualified personnel, the lack of equipment, and the high cost of running these services^21^. As a result, many people who are infected with it do not receive the treatment efficiently. In addition, many people are not aware of the importance of early diagnosis and treatment of infections. This can lead to people delaying seeking treatment, which can make the infection more difficult to treat and increase the risk of complications^22^.

One established risk factor for the recurrence of tuberculosis (TB) is poor adherence to treatment^23^. This disease tends to recur, mostly when it is co-infected with other diseases such as hypertension, diabetes, cardiac illness, asthma, renal disease, pneumothorax, pneumonia, and so on. While undernutrition, smoking, and overcrowding have all been linked to MDR-TB in numerous studies, impoverished populations are also more likely to experience these risk factors^24^. Malnutrition accounts for one-quarter of all tuberculosis infections worldwide^25^. Comorbidities, such as HIV, diabetes mellitus, malignancies, and silicosis, play an important role in susceptibility to tuberculosis infection, in addition to social and economic factors^26^. It has been noted that the risk of MDR/RR-TB was found to be 1.42 times higher, respectively, in HIV-positive individuals than in non-positive individuals^27^.

Additionally, diabetes mellitus is a strong risk factor for the advancement of tuberculosis (TB), and as the prevalence of diabetes mellitus has increased over the past three decades worldwide, today standing at 6.1% the co-occurrence of TB and diabetes mellitus is becoming more widespread^28^. Furthermore, diabetes mellitus raises the risk of MDR/RR-TB by about twofold and is linked to a higher chance of unfavorable treatment outcomes^6^.

Medical professionals and public health experts face a challenge when managing patients with MDR-TB recurrence. Due to the limited treatment options and ongoing risk of close contact, diagnosing, treating, and monitoring MDR-TB cases can be challenging^29^. Mycobacterial culture and drug susceptibility testing facilities are necessary for the diagnosis of MDR-TB cases. Treatment for MDR-TB, which typically consists of three or more effective medications, is also labor-intensive and requires close communication between the patient and the doctor^30^. As a result, it is advised that MDR-TB treatment only be administered in facilities with a mycobacteriology reference center, an isolation facility, and highly qualified medical staff.

Elements necessary for distinguishing recurrences, such as at-risk populations and both the frequency and duration of follow-up^31^, have not been focused on. This study aimed to identify risk factors associated with the recurrence of multidrug-resistant tuberculosis (MDR-TB) among patients at Alert Specialized Hospital. By focusing on at-risk populations and the frequency and duration of follow-up, the research seeks to provide insights that can improve patient outcomes and inform policy decisions. Understanding the prognostic factors related to MDR-TB recurrence is crucial for extending the lives of affected individuals and guiding future interventions. This study contributes to the limited research on MDR-TB recurrence at a specific healthcare facility, emphasizing the importance of tailored approaches in managing this challenging health issue.

## Methodology

### Study design and setting

A retrospective study was employed, and the data was obtained from a cohort of MDR-TB patients enrolled at the hospital, from January 2016 to December 2021. Entry of the data was considered from the date of initiation of MDR-TB drugs. The end time for the patients was when they developed the treatment outcome or when the study time ended in December 2021. The time was measured in months in this study.

This study was conducted at Alert Specialized Hospital, which serves as the highest-level referral hospital for leprosy complications and is recognized by the World Health Organization as an international leprosy training center. Administered by the Ministry of Health of the Federal Democratic Republic of Ethiopia since 2002, the hospital’s mission is to provide specialized medical care, research, and training in the fields of leprosy, TB, HIV/AIDS, tropical dermatology, and other infectious diseases. The hospital aims to improve community well-being and establish itself as a leading center of excellence in medical care, training, and research services in the region.

### Source and study population

All patients diagnosed with MDR TB at Alert specialized hospital between 2016 and 2021 were source population. The subset of MDR TB patients from the source population whose medical records are reviewed and analyzed for the purposes of the retrospective study. Accordingly, a total of 435 MDR-TB patients were our study population.

Inclusion criteria:

- All patients diagnosed with multidrug-resistant tuberculosis (MDR-TB) and registered in the study database or medical records.
- Patients must have complete information available for all the explanatory variables included in the study.
- Patients must have received at least two treatment regimens considered eligible for the study.

Exclusion criteria:

- Patients who started but then discontinued their TB treatment before completion.
- Patients who left the study without ever starting any TB treatment.
- Patients with missing information or incomplete medical records.
- Patients who died due to reasons unrelated to their MDR-TB diagnosis.

### Sample size and sampling procedure of study subjects

This study considered all patients within a specified time period without taking a sample. This approach is often used in retrospective studies or when data for all individuals within a certain timeframe are available and can be analyzed comprehensively^32^. By including all patients who meet the defined criteria and have complete data within the specified time period, researchers can conduct an analysis on the entire population rather than a sample subset.

**Fig. 1:**
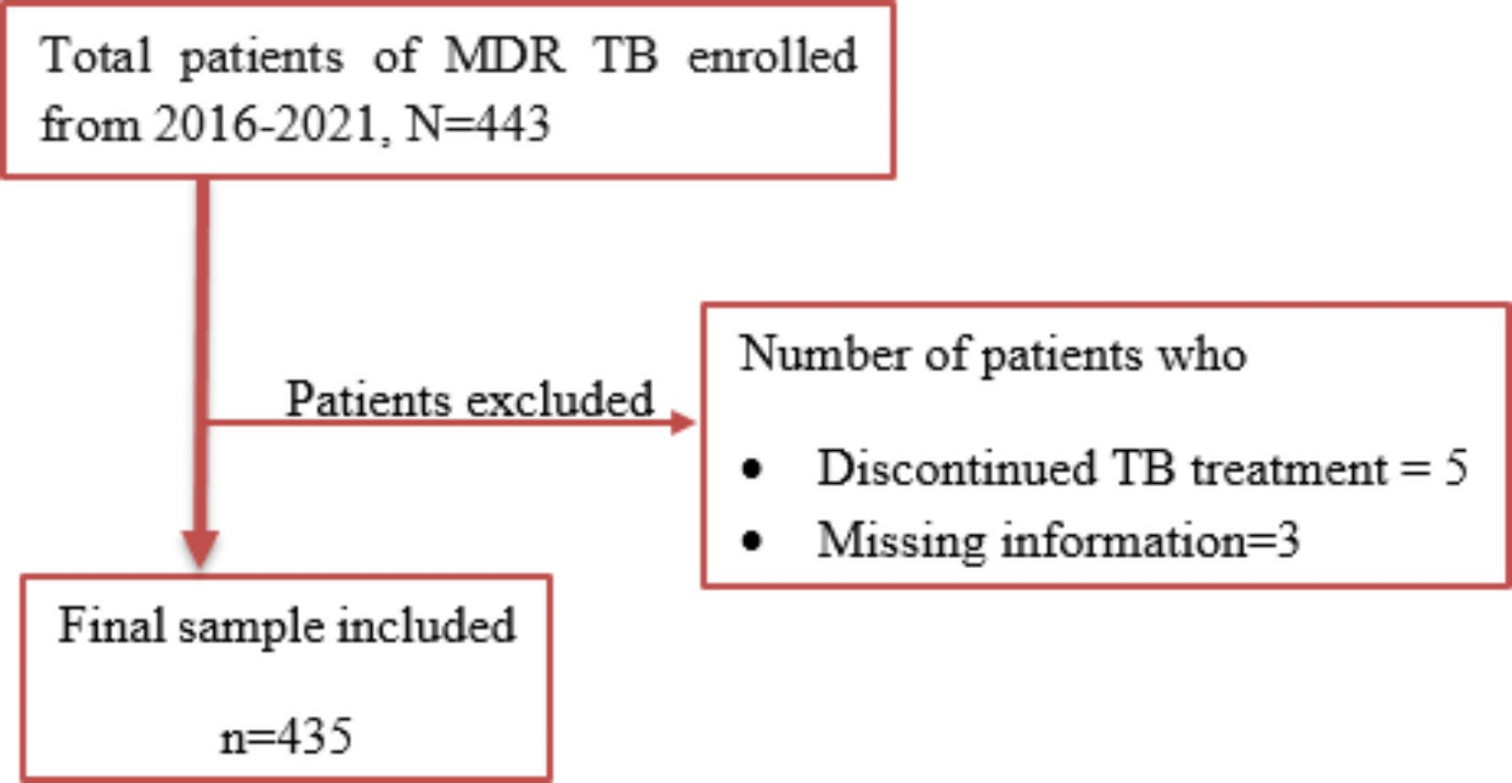
Procedure of obtained patients for the study, Alert Hospital, 2016–2021.

Accordingly, all patients diagnosed with multi-drug-resistant tuberculosis (MDR-TB) who were registered and receiving follow-up care from January 2016 to December 2021 were included in the current study. During this period, a total of 443 individuals were enrolled in the hospital. Each patient in the cohort was retrospectively studied from the initiation of MDR TB diagnosis until the recurrence of disease, death, loss of follow-up, or the end of the study period. Patients who discontinued their TB treatment were not included in the analysis due to the higher risk of negative outcomes. Those with incomplete medical records were also excluded. Eight patients did not meet the criteria and were thus excluded, leaving 435 MDR TB patients for the study (Fig. 1). Survival times were analyzed for these patients, considering those who died from reasons other than TB, those who were alive at the study’s end, or those with missing data after a certain time as censored in the analysis.

### Data extraction and data quality control

The study utilized a data extraction method adapted from the national anti-TB therapy registries to gather information. Data sources included standard anti-TB therapy registries, electronic records, patient medical files, and intake forms. The data extraction process involved five trained nurses working in the hospital’s TB clinics, overseen by an experienced supervisor to maintain data quality. Researchers and the supervisor continuously reviewed checklists for consistency and completeness during the extraction. Information on baseline characteristics such as socio-demographic, clinical, behavioral, and health service details were collected from these sources.

### Variables in the study

- Dependent variable: The response variable was the time to recurrence of the MDR-TB patients, and time was measured in months in this study from diagnosis to the end of the follow-up time.
- Explanatory variables were as follows:
  - Patient demographics include the age of the patient, weight (in kg), education level, and residence.
  - Clinical parameters include the presence of chronic diseases, clinical complications, category of MDR-TB, previous use of anti-drugs, and social drug use.

### Operational definitions

- Drug-resistant TB: TB that is resistant to any first-line anti-tuberculosis drug^8^.
- MDR-TB: a form of tuberculosis caused by bacteria that are resistant to at least two of the most effective first-line anti-TB drugs, isoniazid and rifampin^8^.
- Anti-TB therapy: The treatment regimens consist of a combination of antibiotics designed to target and eliminate the bacteria causing tuberculosis infections^33^.
- Clinical complications: additional medical issues or adverse outcomes that arise as a result of a primary disease or condition, potentially leading to further health challenges and requiring specific management or treatment strategies^34^.
- Survival: The length of time a participant remains alive or free from the event of interest from the start of the study or completion of treatment^35^.
- Censored: A participant whose outcome is unknown or incomplete by the end of the study period^35^.
- Drop out: A participant who actively withdraws or discontinues their participation in the study before the planned end of the study or completion of the intervention^35,36^.
- Loss to follow-up: A participant whose status is unknown or cannot be ascertained by the researchers during the study period^35,37^.

### Dissemination of the finding

The target audience for disseminating the findings from the study on identifying risk factors for recurrent multidrug-resistant tuberculosis (MDR-TB) includes clinicians and public health professionals working in tuberculosis management, as well as policymakers involved in national TB control strategies. The key dissemination channels will involve publication in a peer-reviewed infectious disease or public health journal, presentations at national and international TB/infectious disease conferences, and dissemination through organizational websites, newsletters, and social media.

The dissemination approach will clearly communicate the study design, strengths, limitations, and generalizability of the findings, using effective data visualization techniques to present the key results. The researchers will also engage with national TB programs and the World Health Organization (WHO) to share the findings and their potential impact on MDR-TB management. Furthermore, the researchers will foster collaborations for further research and implementation efforts to build upon these results, with the aim of informing targeted interventions and policies to prevent MDR-TB recurrence.

### Statistical methods

Kaplan–Meier estimates (KM) were used to describe the time to recurrence in MDR-TB patients. Commonly used parametric distributions in survival analysis are the exponential, Weibull, and lognormal^38,39^. The Weibull distribution has a survival function,$\:\:S\left(x\right)=\text{e}\text{x}\text{p}(-\lambda\:x\alpha\:)$, for $\:x\ge\:0$. Here $\:\lambda\:>0$ is a scale parameter, and α > 0 is a shape parameter. The exponential distribution is a special case of the Weibull distribution when α = 1. Because of its historical significance, mathematical simplicity, and important properties, the exponential distribution is one of the most popular parametric models. It has a survival function,$\:\:S\left(x\right)=\text{e}\text{x}\text{p}(-\lambda\:x)$ for $\:x>0$^40^. Although the exponential distribution is popular in some situations, it is restrictive in many real-world applications due to its functional features^41^. Another frequently used distribution to model survival times is the lognormal. If X has a lognormal distribution, then $\:\text{l}\text{n}\left(x\right)$ has a normal distribution. For time-to-event data, this distribution has been popularized not just because of its relationship to the normal distribution but also because it can approximate survival times. The normal and the log-normal distribution are completely specified by two parameters: µ and σ^42^.

In this article, an application of the random effects model for time to recurrence is clustered into regions. The frailty model was used to explain the existence of recurrence differences among patients in different clusters (regions). Observations from the same cluster (region) are supposed to be correlated because they usually share certain unobserved characteristics, like genetic conditions, between them. Disregarding the correlations among the observations may lead to incorrect standard errors in the estimates of parameters of interest. The frailty model was used to introduce random effects in the model to account for association and unobserved heterogeneity^43,44^.

A shared frailty model is considered a mixed model in survival analysis, with group variation (frailty) and individual variation described by the hazard function. A shared gamma frailty with parameters ($\:\frac{1}{\theta\:},\frac{1}{\theta\:}$) is called a one-parameter gamma distribution with a variance parameter$\:\:\theta\:$^39^. With the assumption $\:\gamma\:$= $\:\delta\:\:\:$(necessary for identifiability reasons), the two-parameter gamma distribution turns into a one-parameter distribution$\:{\Gamma\:}(\frac{1}{{\uptheta\:}},\frac{1}{{\uptheta\:}})$. The functional form of the one-parameter gamma distribution is given by:

1 $$\:{f}_{U}\left(u\right)=\:\frac{{u}^{\frac{1}{\theta\:}-1}\text{exp}\left(-u/\theta\:\right)}{{\theta\:}^{\raisebox{1ex}{$1$}\!\left/\:\!\raisebox{-1ex}{$\theta\:$}\right.}{\Gamma\:}\left(\frac{1}{\theta\:}\right)}\:\:\:\:\:\:\:\:\:\:\:\:\:\:\:\:\:\:\:\:\:\:\:\:\:\:\:\:\:\:\:\:\:\:\:\:\:\:\:\:\:\:\:\:\:\:\:\:\:\:\:\:\:\:\:\:\:\:\:\:\:\:\:\:\:\:\:\:\:$$

Thus, the variance and expectation of the frailty variable will be $\:\theta\:$ and 1, respectively.

The inverse Gaussian (inverse normal) distribution was introduced as a frailty distribution alternative to the gamma distribution by^45^. The probability density function of an inverse Gaussian shared distributed random variable with a parameter$\:s\:\theta\:\:>\:0$ is given by:-

2 $$\:fZ\left(Zi\right)=\:\frac{1}{\sqrt{2\pi\:}}{Zi}^{-\frac{3}{2}}{\text{exp}\left(\frac{-\left(zi-1\right)}{2\theta\:zi}\right)}^{2},\theta\:>0,z>0$$

It has a mean of 1 and a variance of *θ*. The parametric shared frailty models were used, assuming the exponential, Weibull, and log-normal distributions for the baseline hazard function. To ensure the accuracy of the final model, the covariates with a p-value ≤ 25% and/or their clinical importance were selected as candidates after conducting a bi-variate analysis for all the covariates in the study. In the final model, only the covariates with a p-value of less than 0.05 were considered significant.

Based on the model selection criteria, we compared the models using AIC; the model with the smallest AIC value was the most efficient model to describe the dataset. Also, by using Cox-Snell residual plots, we checked the goodness of fit of the model; if the plot shows that the line related to the Cox-Snell residuals is closely related to the line through the origin, this indicates that the model is well-fitting the data set. The data was analyzed using R version 3.5.3.

## Results

The descriptive summaries of baseline categorical covariates are given in Table 1. A total of 435 MDR-TB patients were included in the study during data collection. Out of this, 34.3% were recurrences. The overall median recurrence time of MDR-TB patients was 12 months [95% CI 11, 14]. From the results, we see that males (23.7%) had a recurrence due to MDR-TB and females (10.6%). MDR-TB recurrence was found in 24.6% of urban residents and 9.7% of rural residents. The MDR-TB recurrence among non-users, khat users, alcohol users, smokers, and combinations of any two or more social drug users was 14%, 0.5%, 14.7%, 3.7%, and 1.4%, respectively. MDR-TB patients who were HIV-negative (22.3%) The median and standard deviation [median ± SD] of the age and weight of patients were 40 14.56 years and 55 6.91 kg, respectively (Table 1).

**Table 1:** Descriptive summary of the patient’s characteristics for the MDR-TB.

Covariates	Patients		Recurrence		Median in month (95% CI)
	Number	Percent	Number	Percent	
Sex: Male	347	79.8	103	23.7	12 (11, 13)
Female	88	20.2	46	10.6	7 (6, 10)
Education level: illiterate	80	18.4	19	4.4	12 (11, 14)
Primary	133	30.6	56	12.9	9 (8, 10)
Secondary	74	17.0	26	6.0	8 (7, 12)
Diploma	62	14.3	15	3.4	11 (9, 13)
Degree and above	86	19.8	33	7.6	9(4, 12)
Region: Addis Ababa	156	35.9	45	10.3	9 (8, 10)
Oromia	105	24.1	34	7.8	10 (11, 13)
SNNP	71	16.3	27	6.2	11 (9, 12)
Amhara	54	12.4	24	5.5	12 (9, 14)
Other	49	11.3	19	4.4	12 (9, 15)
Residence: Urban	347	79.8	107	24.6	13 (12, 14)
Rural	88	20.2	42	9.7	12 (9, 14)
Social drug: Non-user	166	38.2	61	14.0	12 (8, 17)
Khat use	65	14.9	2	0.5	12 (7, 16)
Alcohol use	149	34.3	64	14.7	13 (8, 15)
Smoker	35	8.0	16	3.7	12 (4, 13)
Two or more social	20	4.6	6	1.4	10 (8, 14)
HIV status: Negative	297	68.3	97	22.3	11 (8, 12)
Positive	138	32.7	52	11.9	11 (9, 18)
Chronic-Disease					
Hypertension	152	34.9	52	12.0	12 (9, 14)
Diabetes	129	29.7	35	8.0	13 (9, 18)
Cardiac illness	69	15.9	24	5.5	12 (12, 14)
Asthma	37	8.5	16	3.7	9 (4, 11)
Renal disease	27	6.2	8	1.8	12 (8, 15)
two or more chronic	21	4.8	14	3.2	8 (5, 9)
Category: Pulmonary	365	83.9	134	30.8	11 (9, 12)
Extra-Pulmonary	70	16.1	15	3.4	13 (10, 14)
Previous TB: Yes	89	20.5	47	10.8	9 (7, 10)
No	346	79.5	102	23.4	12 (10, 13)
Clinical-complication: No	175	40.2	72	16.6	13 (8, 16)
Pneumothorax	72	16.6	1	0.2	10 (7, 11)
Pneumonia	132	30.3	42	9.7	12 (10, 14)
Hemoptysis	35	8.0	23	5.3	9 (6, 10)
Corpulmonale	8	1.8	4	0.9	8 (6, 11)
Combination of any	13	3.0	7	1.6	8 (5, 9)

Source: Alert Specialized Hospital, Ethiopia; from January 2016 to December 2021.

The covariates that were not significant at the 25% level in bivariate analysis were not involved in multivariable analysis. The AIC value of the Weibull–Inverse–Gaussian model was 899.300, which is the minimum among all the other models. Therefore, the Weibull-Inverse-Gaussian model was the most effective model to define the MDR-TB patients’ dataset (Table 2).

**Table 2:** AIC values of the parametric frailty models.

Baseline distribution model		AIC
Weibull	Inverse-Gaussian	899.300
	Gamma	920.867
Exponential	Inverse-Gaussian	1020.616
	Gamma	1019.900
Lognormal	Inverse-Gaussian	976.008
	Gamma	964.911

Source: Alert Specialized Hospital, Ethiopia; from January 2016 to December 2021.

*AIC* Akaike’s Information Criteria.

After the bivariate analysis, we observed that age, weight, education level, residence, social drug use, clinical complication, chronic diseases, category of MDR-TB, and previous anti-drug history covariate variables were significantly related to identifying risk factors for multiple drug-resistant tuberculosis recurrence at 25% level of significance in the entire models used, except sex, and HIV status (Table 3). The confidence intervals of the acceleration factors for covariates do not comprise one in all models, at a 5% level of significance. This indicates that they are significant risk factors for multiple drug-resistant tuberculosis recurrence. However, the sex and HIV status were not significant for the risk factors of multiple drug-resistant tuberculosis recurrence, according to all the models at a 25% level of significance. Hence, based on this outcome, it is well to ignore the sex, and HIV status covariate and then shall do our multivariable analysis by using the significant factors. Then, in multivariable analyses, we performed by considering all covariate variables that are significant with corresponding to the bivariate models at a 25% level of significance.

Once the best model was identified, a separate analysis was conducted. All variables were deemed significant when the p-value was below 5%. According to the Weibull-Inverse-Gaussian model, the statistically significant variables included age, weight, social drug use, chronic diseases, clinical complications, previous anti-drug history, educational level, residence, and category of MDR-TB of the patients. Specifically, weight increment, smokers and alcohol users, clinical complications of hemoptysis and pneumonia, patients with pulmonary disease who had a history of previous anti-drug treatment, and being rural residents significantly reduced the time to recurrence of patients. Conversely, patients with an education status of degree & above and an increased age significantly extended the time to recurrence of patients (Table 3).

An acceleration factor greater than 1 indicates an extension in the time to recurrence. For patients identified as social drug users, specifically smokers and alcohol users, the acceleration factors were 0.045 and 0.631, respectively. This suggests that smokers and alcohol users experienced a shorter time to recurrence compared to non-drug users. Additionally, patients who experienced chronic complications such as hemoptysis showed an estimated acceleration factor of 0.041, accompanied by a significant p-value (*p* = 0.001; 95% CI 0.006, 0.269). This finding indicates that patients with hemoptysis complications had a quicker time to recurrence compared to those without clinical complications.

Similarly, patients with pneumonia had an estimated acceleration factor of 0.564, accompanied by a small p-value (*p* = 0.016; 95% CI 0.354, 0.898). This suggests that patients with pneumonia complications had a quicker time to recurrence compared to those without clinical complications. Different categories of MDR-TB patients exhibited significantly varied times to recurrence.

When it comes to residence, patients residing in rural areas experienced a faster time to TB recurrence compared to urban patients (ɸ = 0.163; 95% CI 0.042, 0.636).

Regarding, patients with primary education status (ɸ = 0.436; 95% CI 0.207, 0.917) and diploma (ɸ = 0.365; 95% CI 0.141, 0.944) had a quicker time to recurrence than the reference category, while those with a degree or above (ɸ = 3.525; 95% CI 1.481, 8.386) had a longer time to recurrence than the reference group.

As the patient’s age increases, the time to TB recurrence lengthens (ɸ = 1.021; 95% CI 1.005, 1.037). Additionally, as the patient’s weight increases, the time to TB recurrence shortens (ɸ = 0.944; 95% CI 0.908, 0.982).

Moreover, patients with a history of previous MDR-TB (ɸ = 0.106; 95% CI 0.539, 0.625) had a quicker time to recurrence than those without a history of TB.

Finally, regarding the presence of chronic disease, individuals who developed diabetes (ɸ = 0.442; 95% CI 0.307, 0.837) had a faster time to recurrence than the reference group (Table 3).

**Table 3:** Results of bivariate analysis and multivariable analysis using the Weibull- inverse gaussian frailty model.

Variables	Categories	Crude estimates		Adjusted estimates			
		Estimate	ɸ	Estimate	*p*-value	ɸ	95% CI for ɸ
Age	Continuous	0.022	1.022*	0.021	0.008	1.021	[1.005 ,1.037]
Sex: Male	Female	1.12	3.064				
Weight	Continuous	− 0.59	0.554*	− 0.057	0.005	0.944	[0.908 ,0.982]
Education level: Illiterate	Primary	− 0.78	0.458*	− 0.830	0.029	0.436	[0.207 ,0.917]
	Secondary	− 0.520	0.594*	− 0.540	0.098	0.582	[0.307 ,1.105]
	Diploma	− 1.005	0.366*	− 1.009	0.038	0.365	[0.141 ,0.944]
	Degree and above	1.280	3.596*	1.260	0.004	3.525	[1.481 ,8.386]
HIV status: Negative	Positive	− 0.513	0.599				
Social drug: Non-use	Smoker	− 3.083	0.045	− 3.092	0.001	0.045	[0.007 ,0.301]
	Alcohol use	− 0.458	0.632*	− 0.460	0.037	0.631	[0.409 ,0.973]
	Khat use	− 0.315	0.729	− 0.317	0.378	0.728	[0.360 ,1.474]
	Comb. of ≥2	− 0.551	0.576*	− 0.554	0.35	0.575	[0.180 ,1.837]
Chronic diseases: Hypertension	Diabetes	− 0.819	0.440*	− 0.815	0.027	0.442	[0.307 ,0.837]
	Cardiac illness	0.367	1.443	0.362	0.242	1.436	[0.783 ,2.632]
	Asthma	0.178	1.194*	0.175	0.573	1.191	[0.648 ,2.188]
	Renal Diseases	0.560	1.750*	0.558	0.247	1.747	[0.680 ,4.492]
	Comb. of ≥2	− 0.268	0.764	− 0.274	0.315	0.760	[0.446 ,1.297]
Clinical: No-complication	Hemoptysis	− 3.184	0.041*	− 3.187	0.001	0.041	[0.006 ,0.269]
	Pneumonia	− 0.570	0.565*	− 0.573	0.016	0.564	[0.354 ,0.898]
	Pneumothorax	− 0.308	0.734	− 0.310	0.302	0.733	[0.408 ,1.320]
	Corpulmonale	0.361	1.434	0.364	0.522	1.439	[0.472 ,4.381]
	Comb. of ≥2	− 0.043	0.957	− 0.047	0.927	0.954	[0.348 ,2.617]
Previous TB: No	Yes	− 2.210	0.109*	− 2.240	< 0.001	0.106	[0.539 ,0.625]
Residence: Urban	Rural	− 1.810	0.164*	− 1.814	0.009	0.163	[0.042 ,0.636]
Category: Extra-Pulmonary	Pulmonary	− 2.187	0.112*	− 2.195	< 0.001	0.112	[0.043 ,0.292]

*Significant at 25% at univariable level. θ = 0.159, (*p* = 0.018), τ = 0.065, λ = 0.040, SE (0.04), ρ = 2.491, SE (0.183), AIC = 899.300, $\:{\uptheta\:}\:$= Variance random effect,$\:\:{\uptau\:}$ = Kendall’s Tau,$\:\:{\uplambda\:}\:$= lambda, $\:\:{\uprho\:}\:\:$=shape, ɸ = Accelerated factor.

Source: Alert Specialized Hospital, Ethiopia; from January 2016 to December 2021.

### Kaplan–Meier (KM) curve of significant variables of MDR-TB patients’ recurrence

Descriptive graphs of survivor function would be used for the determination of comparing the event experiencing time of two or more groups and the survival quantities of covariates to describe the survival experience of an individual at specific times.

**Fig. 2:**
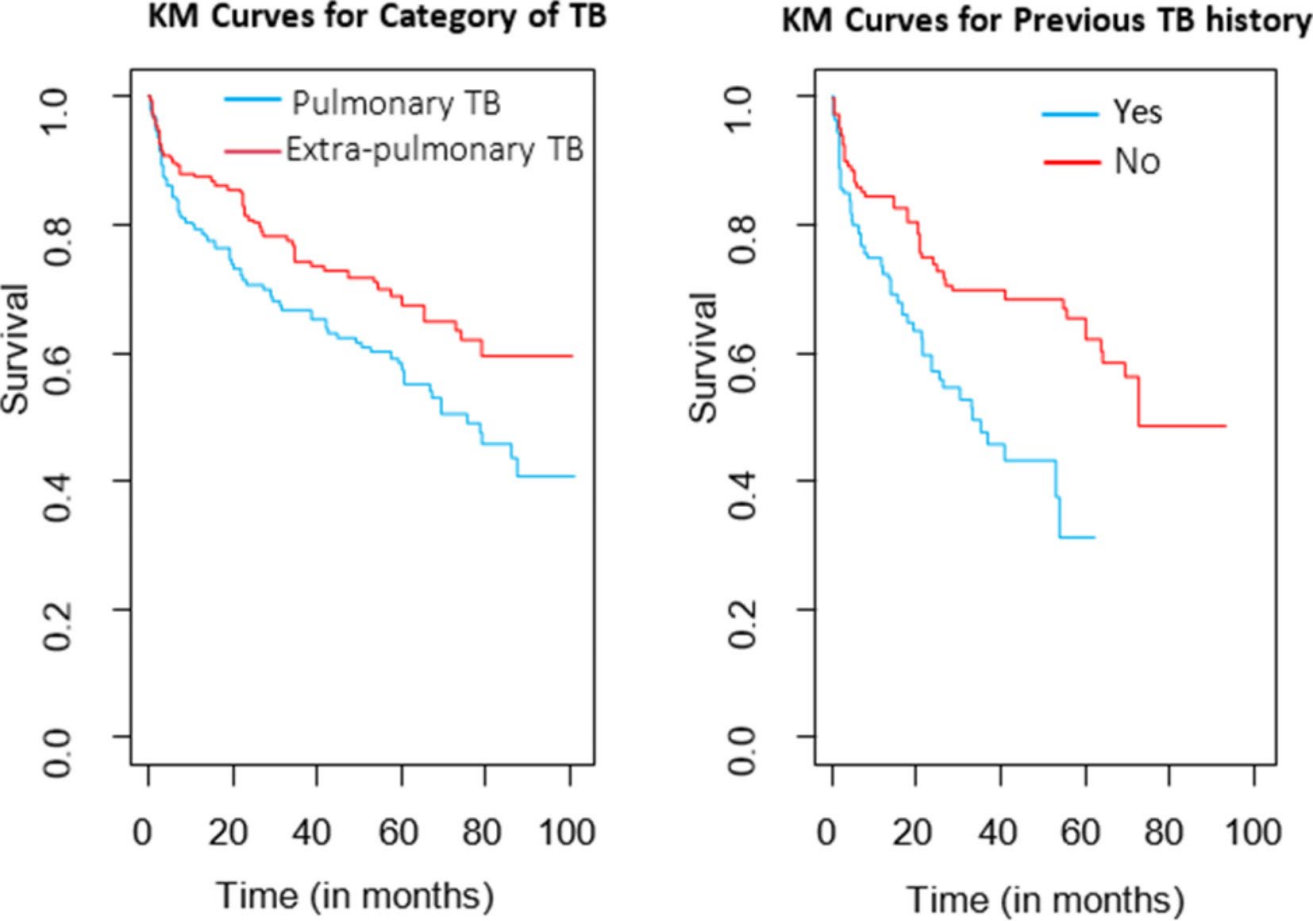
KM curve of MDR-TB patients’ recurrence for the type of TB (Left) and previous TB history (Right).

In Fig. 2, the survival function plotted against survival time illustrates the recurrence of MDR-TB across different categories. The categories of MDR-TB showed significance at a 5% level in frailty models when compared to the reference group. The gap between the two curves highlights the survival distribution in identifying risk factors for recurrent multidrug-resistant tuberculosis across categories. The KM curve compares the survival of patients with Pulmonary TB and Extra-pulmonary TB on the left, while the other curve compares patients with and without a previous history of TB on the right. These curves depict the survival probability over time in months, with the Pulmonary TB and previous TB history groups generally exhibiting better survival rates compared to their counterparts. The discrepancies in the survival curve reveal that patients categorized under the pulmonary group have longer recurrence times compared to those in the extra-pulmonary group.

**Fig. 3:**
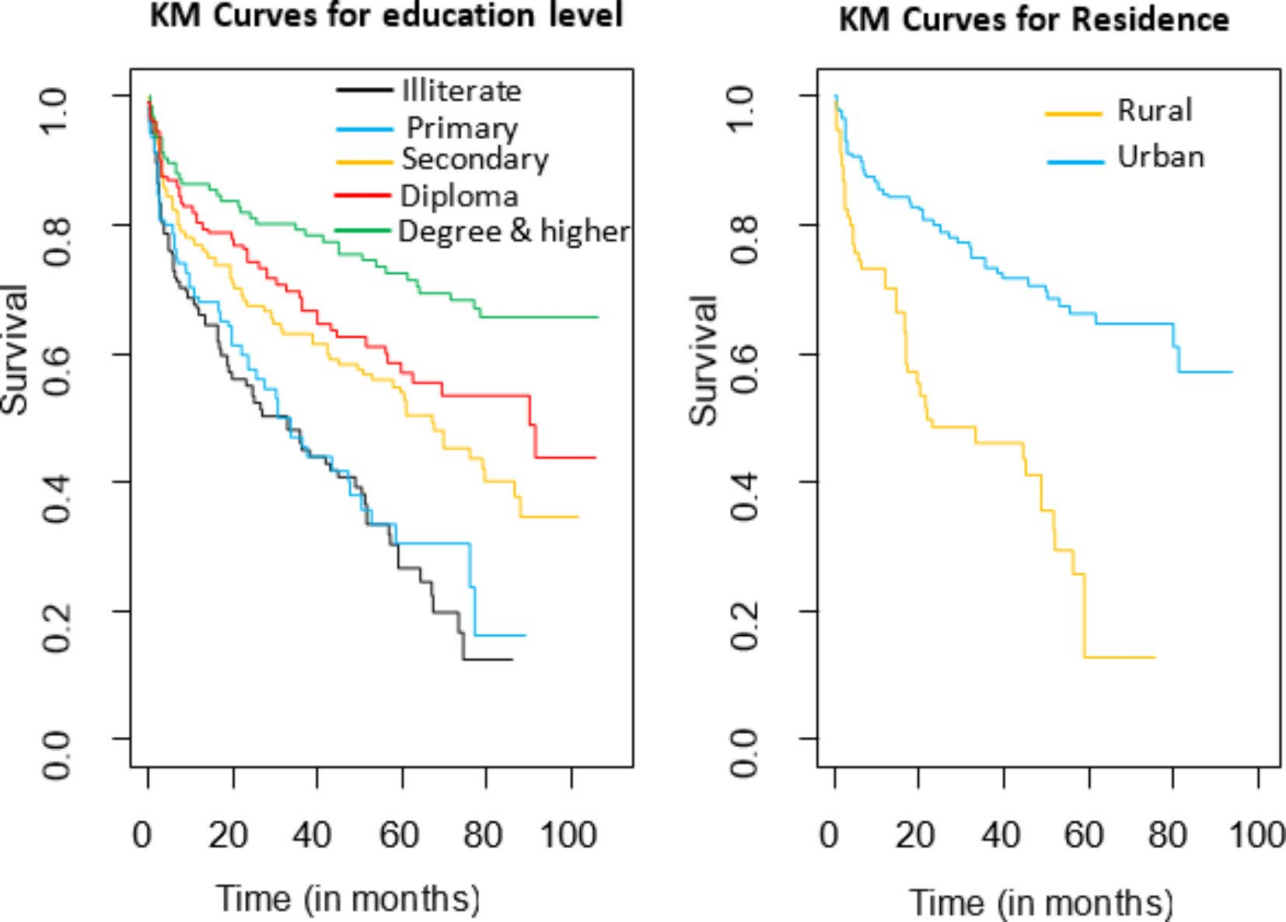
KM curve of MDR-TB patients’ recurrence for education level (left) and residence (right).

As illustrated in Fig. 3, on the left side, the graph titled “KM Curves for education level” displays five lines representing different education levels: Illiterate, Primary, Secondary, Diploma, and Degree & higher. Each line depicts the survival probability over time in months, with “Degree & higher” starting at the highest survival rate and “Illiterate” at the lowest. On the right side, the graph titled “KM Curves for Residence” shows two lines for Rural and Urban residences. The urban survival curve consistently remains above the rural curve, indicating a higher survival rate for urban residents compared to rural residents over the observed time period.

**Fig. 4:**
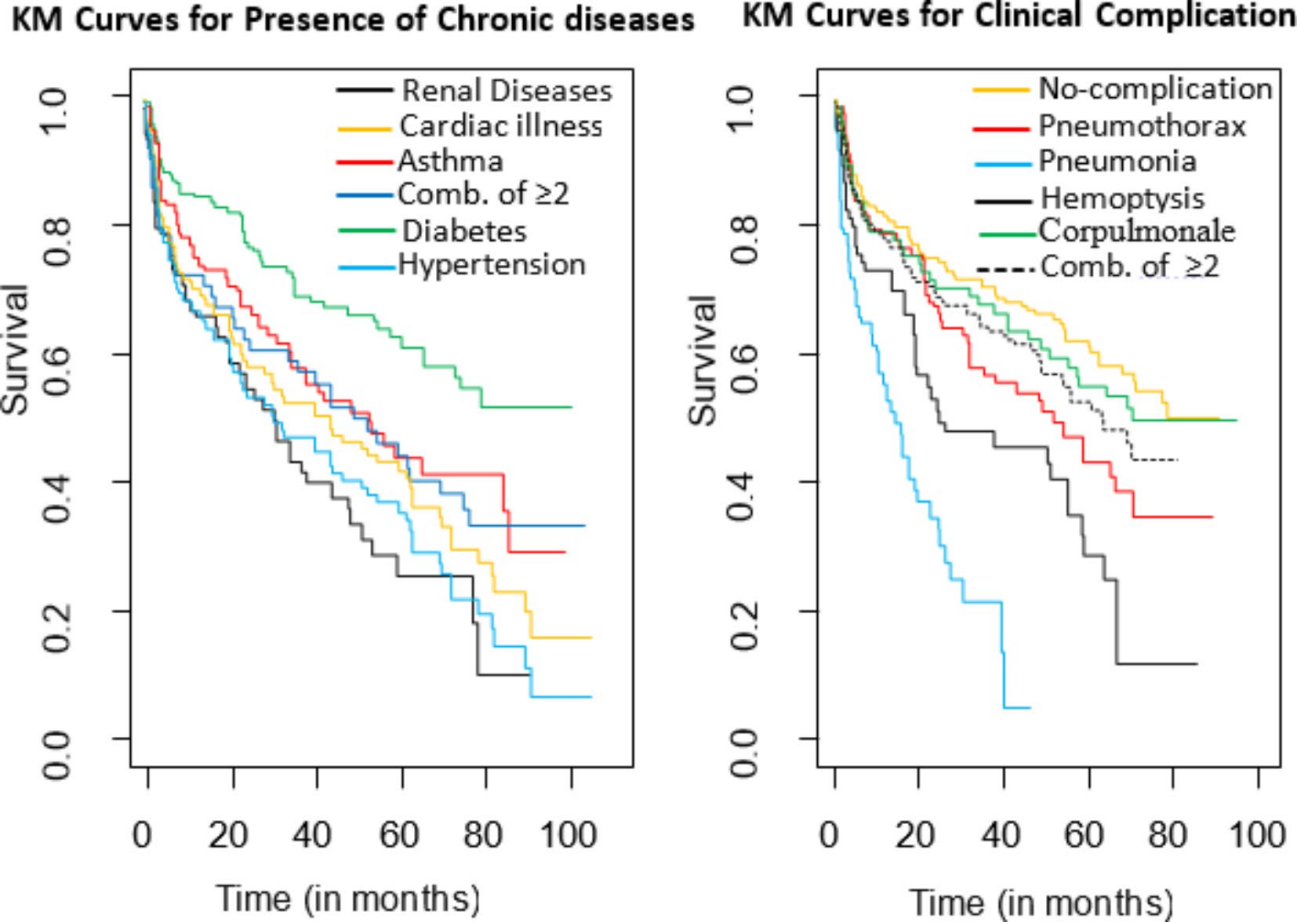
KM curve of MDR-TB patient recurrence by presence of chronic diseases (left) and clinical complication (right).

As shown in Fig. 4, the survival function plotted against survival time visualizes the recurrence of MDR-TB across various chronic diseases (on the left) and clinical complications (on the right). In frailty models, both the presence of chronic diseases and the existence of clinical complications exhibited a significant impact on the recurrence of MDR-TB at a 5% level of significance.

On the KM curve for chronic diseases (on the left), the diabetes curve stands out prominently from the others on the left, not intersecting with them, indicating significant disparities and prolonged survival compared to other categories. Similarly, on the KM curve for clinical complications (on the right), Pneumonia and Hemoptysis demonstrate significant differences, but with comparatively shorter survival times as visually depicted on the graph.

**Fig. 5:**
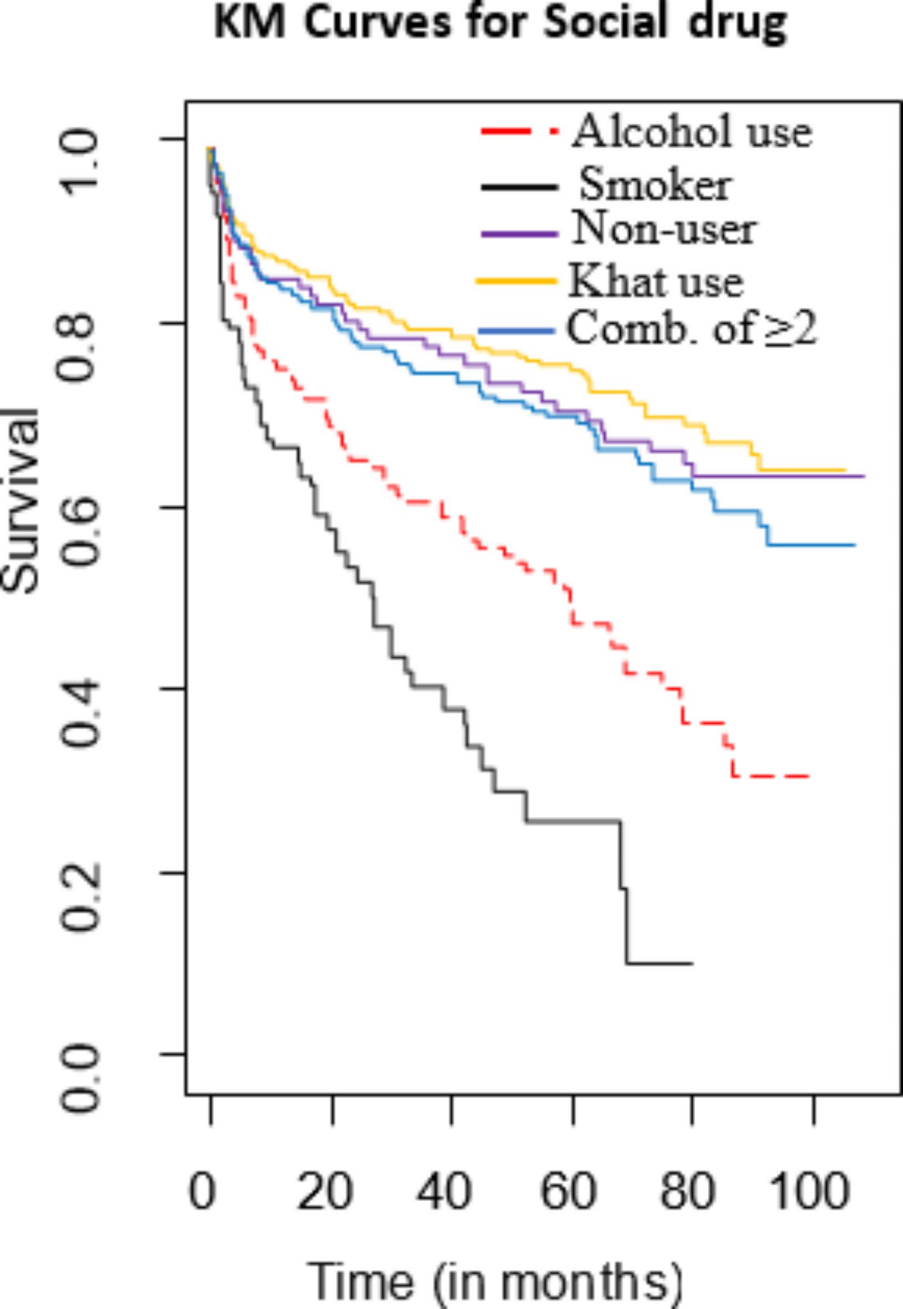
KM curve of MDR-TB patient recurrence by social drug use.

As depicted in Fig. 5, the survival function illustrates the recurrence of MDR-TB over time across different social drug use groups. According to frailty models, social drug use significantly influences MDR-TB recurrence at a 5% significance level. On the Kaplan–Meier curve for social drug use, alcohol consumption and smoking notably diverge from other categories, distinctly not overlapping with the reference group (non-users). This observation suggests significant disparities in recurrence rates. Furthermore, both alcohol use and smoking are associated with shorter survival times compared to other categories, as evidenced by their curves consistently lying below the others on the plot.

### Overall survival curve of MDR TB patients

**Fig. 6:**
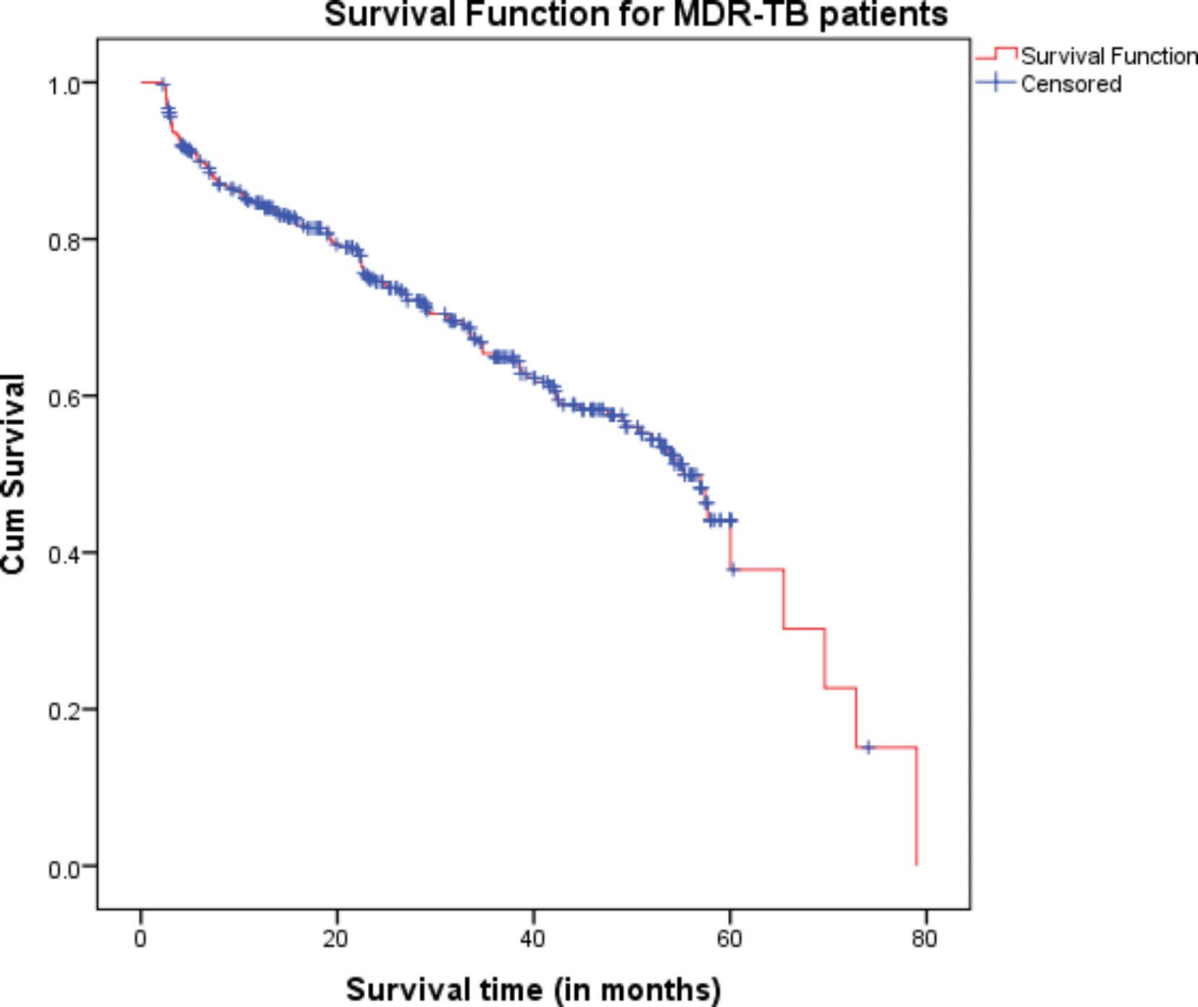
Overall survival curve for MDR-TB patients.

The overall survival curve for MDR TB patients shows the proportion of individuals who are still alive at different points in time after diagnosis. A declining curve indicates a decrease in survival rates over time, suggesting that the prognosis for individuals with MDR TB may be getting worse as time goes on (Fig. 6).

**Fig. 7:**
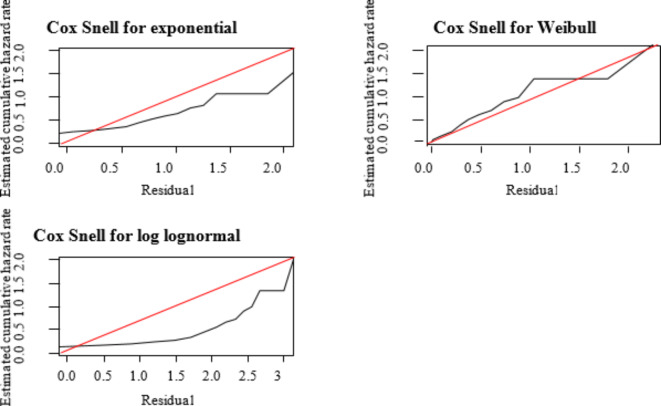
Cox–Snell residuals for parametric models.

The shape of the parameter value in the Weibull-Inverse Gaussian frailty model was 2.491, which indicates that the value is greater than unity, which shows the shape of the hazard function is unimodal. It ascends to some point and then descends. The variability (heterogeneity) in the population of clusters estimated by the best model is 0.159 with *p* = 0.018 and the dependence within clusters is about 0.065. There were recurrence differences among patients from region to region in Ethiopia. The Cox–Snell residuals are an exceptional method to study how well the model fits the data. The Cox–Snell plot of the Weibull distribution seems more linear than other distributions. This shows that the Weibull distribution describes our data well (Fig. 7).

**Fig. 8:**
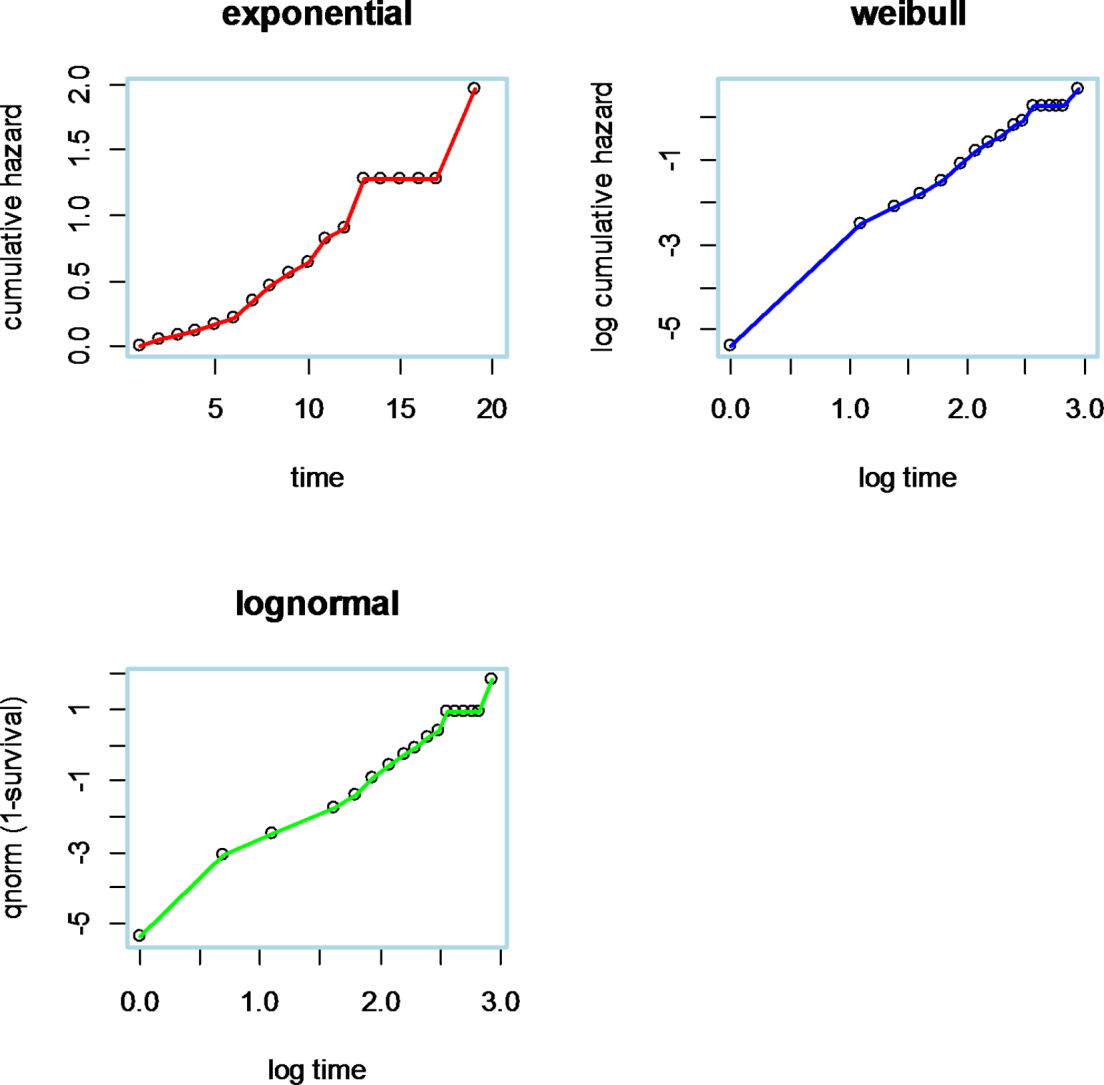
Parametric assumption checking models.

The plot shows that the line related to the Cox–Snell residuals of the Weibull model was nearest to the line through the origin; this model defines the MDR-TB data set well. This outcome consolidates the result from the log cumulative hazard plot (Fig. 8).

**Fig. 9:**
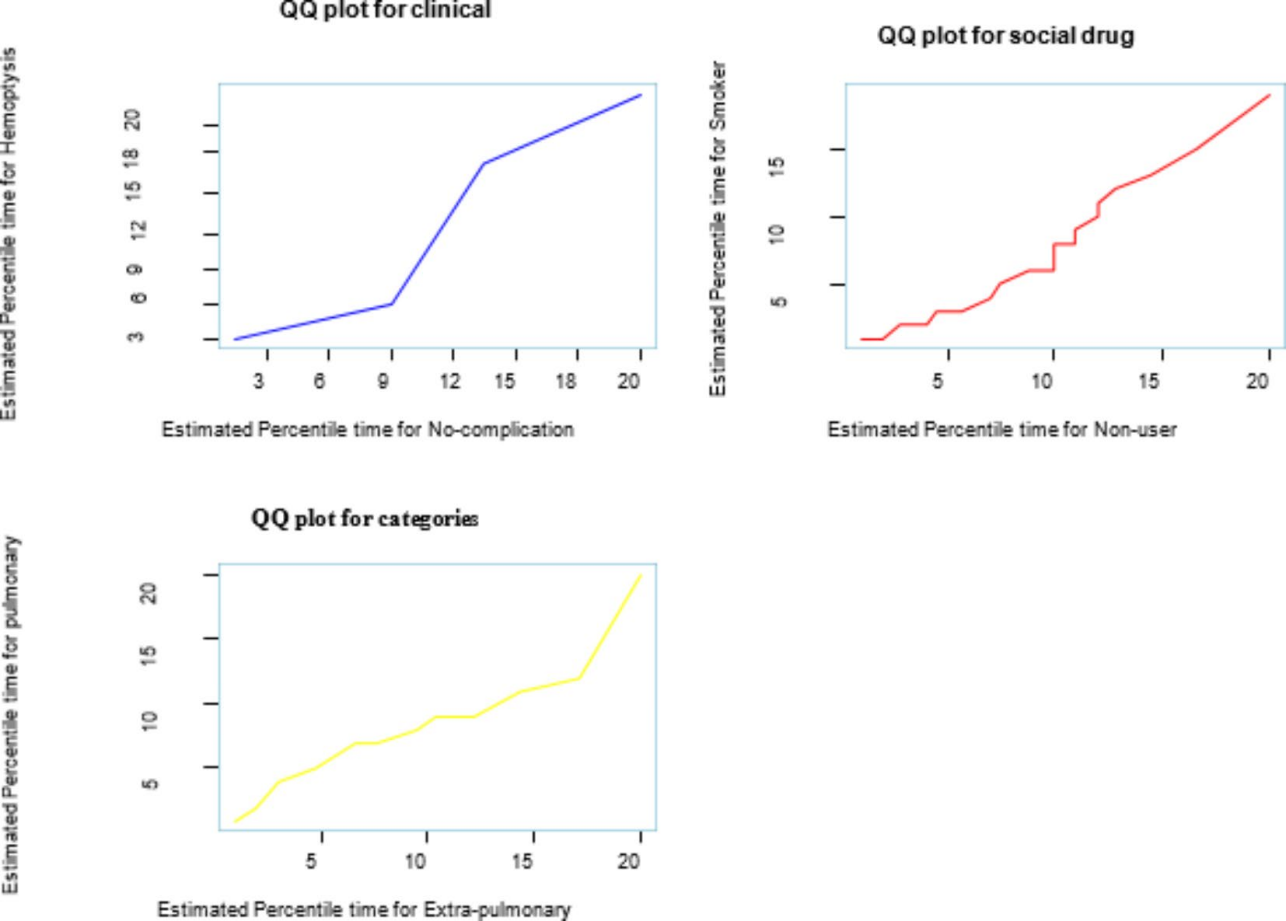
Q–Q plot to check the adequacy of the accelerated failure time model.

A quantile–quantile plot is made to check if the accelerated failure time delivers an adequate fit to the data by using two different groups of observations. The adequacy of the model was checked by using different significant variables. The accelerated failure time appears to be the best way to describe the recurrence time of the MDR-TB dataset (Fig. 9).

## Discussion

This study highlights the significant impact of frailty effects based on region on the recurrence of multi-drug-resistant tuberculosis (MDR-TB). This is supported by study from northern Ethiopia^46^. This might be due to regional variations, including distinct epidemiological profiles and healthcare infrastructure that play a crucial role in influencing treatment outcomes and recurrence rates. Also, this study found that the median time for MDR-TB recurrence was 12 months.

This study reported that out of 34.3% of patients’ experienced MDR-TB recurrence. This recurrence is significantly higher than the global pooled recurrence rate of 16.9% reported in the systematic review and meta-analysis by^47^. This in agreement with study reported nearly lower rate, 29.5% of recurrence from study in China^48^. Furthermore, another study reported a much lower MDR-TB recurrence rate of 5.2% from Russia^49^, in contrast to the current study’s recurrence rate. The discrepancy in recurrence rates between the two studies highlights the regional variations in the burden of MDR-TB and the challenges faced in different healthcare settings. The higher rate of MDR-TB recurrence in the initial study setting can be attributed to factors such as limited access to comprehensive treatment, emergence of new drug resistance due to incomplete treatment, and socioeconomic or environmental conditions that contribute to the persistence of the disease within certain communities. Moreover, another study from Taiwan reported even lower recurrence rate with 3% experienced MDR-TB recurrence during a follow up period of 4.8 years^50^. This might due to lower follow-up period.

Patients who were non-users of social drugs had a longer time to MDR-TB recurrence compared to those who were social drug users. The study is consistent with the studies conducted by^51^ alcohol consumption rises hazard of MDR-TB by the rate of 4.53 as compared to non-alcohol users. This finding is also in agreements with similar studies like^52,53^. In addition studies from Atlanta, Georgia^54^, Hong Kong, Pakistan, and the USA^55–57^ reported similar results. This study also agrees with the study conducted in Iran by^58^. This might be due to the fact that using drugs like alcohol and smoking was consistently indicated to increase the incidence of recurrence in patients with MDR-TB, facilitate high-imposed infections, and concession their immunities, which in turn shorten the survival time to recurrence for the individuals. This finding is consistent with the study conducted by^59^.

This study showed that the risk of recurrence for MDR-TB patients with different clinical complications (like pneumonia complications and hemoptysis complications) had a shorter survival time relative to MDR-TB patients with non-clinical complications. This might be due to the fact that pre-existing infections can be further complicated by newly developing diseases. These newly emerging diseases minimize individual immunizations, which can shorten their recurrence time. This study showed that the duration of the recurrence of MDR-TB patients with diabetes had shorter survival times to recurrence than the reference group. This finding was similar to the previous study by^52^ and also in the United Kingdom^60^, and the study was also agreeable with those conducted in Chain and Uzbekistan^61,62^, respectively, and in the USA by^55^. This indicates that the cause is an emerging epidemic in developing countries where the prevalence of MDR-TB is increasing. Although it is known that chronic diseases increase the risk of MDR-TB recurrence, it has not been well studied. This might also be due to co-morbidity, which is a risk factor for different diseases like infectious and non-infectious, which could reduce the duration of the individual’s survival time for recurrence.

The study found that age was a significant covariate for the recurrence of MDR-TB in patients. This finding was similar to those conducted in Ethiopia^63^. Atlanta, Georgia^54^, and China^61,64^. This might be due to the fact that as the age of the patients increases, their recurrence time decreases. This is consistent with the study conducted by^58^. However, this finding is inconsistent with the study conducted by^65^.

In this finding, the weight increment is one risk factor for recurrence in the patients. Then this study is consistent with the last study in Brazil by^66^ and Atlanta, Georgia, by^67^. This might be because increased weight exposes individuals to several diseases, like chronic degenerative infections, which reduce their recurrence.

In this study, patients who had primary had a faster survival time for TB recurrence, and those who had degrees & above had a longer survival time compared with the reference group. This finding agreed with the studies done in Brazil^66^ and China^48,64^. This agreement may be attributed to the possibility that individuals with lower levels of education might have less awareness of their health issues. However, this finding disagrees with the study conducted by^65^, which revealed education status has no effect on TB recurrence. This discrepancy could be due to differences in the specific patient populations, methodologies, or statistical analyses used in the studies. Additionally, variations in healthcare systems, cultural factors, and access to resources in different regions as their study was conducted at southern part of Ethiopia could also contribute to differing results across studies.

In this study, we consider that the types of tuberculosis are also a risk factor for the recurrence time of the patients. This finding is consistent with the studies conducted in England and Wales, Singapore, and by van der Heijden^68,69^, respectively. In our study, the patients who had a previous history of TB were the prognostic factors for recurrence in the MDR-TB patients. This study agrees with the findings that were conducted in Vietnam by Bestrashniy et al.^70^ and Abera et al.^59^.

According to the current study, HIV status does not significantly impact the recurrence of multidrug-resistant tuberculosis (MDR-TB). This means that people living with HIV and those without HIV have similar chances of experiencing MDR-TB recurrence. However, this finding is inconsistent with different studies reported in the literature. For example, a study reported in^71^ found that the pooled odds of MDR-TB were 1.42 times higher in HIV-positive patients compared to HIV-negative patients. Similarly, males and females had no significant difference in the recurrence of multidrug-resistant tuberculosis (MDR-TB). However, some previous studies have suggested that men may be more likely to experience MDR-TB recurrence compared to women^23,55^. This gender-based difference in MDR-TB recurrence rates could potentially be attributed to biological factors, such as variations in immune response, or social factors, like disparities in access to healthcare.

## Conclusions

The variables that had a significant factor in the recurrence of MDR-TB patients were age, weight, social drug usage, clinical complications, MDR-TB category, education level, previous TB history, and place of residence. Weight of MDR-TB patients, secondary and degree education status, smokers, alcohol users, diabetic patients, previous history of TB, clinical complications of hemoptysis and pneumonia, pulmonary TB, and rural residents had a shorter survival time than others. Those with different levels of education and ages, on the other hand, had a longer survival time. The recurrence rate of MDR-TB patients varied by region at Alert Specialized Hospital in Addis Ababa, Ethiopia. The Weibull-Inverse-Gaussian shared frailty model was selected as the best model for forecasting the time of recurrence of MDR-TB patients in the study area.

### Recommendations

The concerned bodies and the Ministry of Health of Ethiopia should focus on significant variables of TB recurrence, such as weight gain and social drug use. According to WHO guidelines, the diagnosis and management of TB recurrence should be improved, and operational research studies should be conducted to provide answers to some open questions.

### Limitations and strengths of the study

In this paper, the effects of MDR-TB interventions, nutritional and laboratory information, vaccination on initial CXR, culture conversion before initiating SLD, and sputum smear positivity at the time of diagnosis were not considered, which a limitation of the study in general and a prediction model in particular.

## Data Availability

The data used to support the findings of this study are available from the corresponding author upon request.

## Abbreviations

TB: Tuberculosis
HIV: human immunodeficiency virus
SLD: Second-line drug resistance
MDR-TB: Multidrug Resistant Tuberculosis
CXR: Chest radiography
